# Determinants of quality of life in French nursing home residents across cognitive levels: a comparative study using convergent mixed-methods

**DOI:** 10.1186/s12877-024-05226-4

**Published:** 2024-07-30

**Authors:** Christophe Cousi, Valérie Igier, Bruno Quintard

**Affiliations:** 1 Centre for Studies and Research in Psychopathology and Health (CERPPS), CLESCO ED 326, University of Toulouse Jean-Jaurès, Toulouse, France; 2 LabPsy Psychology Laboratory UR 4139, University of Bordeaux, Bordeaux, France

**Keywords:** Quality of life, QoL, Dementia, Alzheimer’s disease, QoL-AD NH, Predictors, Factors, Nursing Homes, EHPAD, Mixed Methods

## Abstract

**Background:**

The quality of life (QoL) of nursing home residents is multifaceted and influenced by relationships, health, and activities, as per research in international literature. However, studies exploring QoL predictors considering varying cognitive impairment levels are limited in the French context. This study examined the impact of sociodemographic factors and cognitive impairment on the QoL in Alzheimer’s Disease Nursing Homes (QoL-AD NH) scale scores among French nursing home residents. It further identified predictors through responses to qualitative semi-structured interviews. These elements were integrated and compared to understand more comprehensively the multifaceted determinants influencing residents’ QoL.

**Methods:**

This mixed methods study used a cross-sectional convergent design, and quantitative and qualitative studies were carried out simultaneously. Using a generalised linear model and Kruskal–Wallis tests, the quantitative strand (*N* = 151) measured QoL with the QoL-AD NH scale and examined sociodemographic predictors of QoL. The qualitative strand (*N* = 78) involved semi-structured interviews with residents across four levels of cognitive functioning (no, mild, moderate, and severe impairment) and explored their QoL determinants through thematic analysis. Both strands were then integrated and analysed.

**Results:**

Mild cognitive impairment and depression negatively predicted QoL-AD NH scores. For specific items, residents with mild cognitive impairment had lower “Ability to keep busy daily” and “Current life in general” scores than residents without cognitive impairment. Qualitatively, family relationships were indispensable for QoL across groups, but those with mild cognitive impairment complained about a lack of activities in nursing homes. The analysis identified convergent predictors and enriched our understanding of daily occupation. Theory comparisons revealed assessment limitations in psychological well-being.

**Conclusions:**

A mixed approach provided a nuanced understanding of QoL, highlighting vulnerable groups and areas for improving assessment. Combining the results from standardised instruments with semi-structured interviews allowed us to capture a fuller range of experiences. The findings suggest a need to reconsider QoL assessment tools for nursing home residents and policies to address their needs regardless of their cognitive levels. They highlight the value of mixed methods for researching this multifaceted field.

**Supplementary Information:**

The online version contains supplementary material available at 10.1186/s12877-024-05226-4.

## Background

Quality of life (QoL) is a pivotal concept in healthcare, offering crucial insights into the well-being and satisfaction of individuals in various settings, including nursing homes. Understanding the conceptual foundations and measurement approaches of QoL is essential for comprehensively examining its implications for nursing home residents with and without Alzheimer’s disease.

While the WHO has offered a general definition of QoL, Lawton’s model, despite its age, remains the most commonly adopted conceptualisation of QoL in dementia patients, even if it is not specific to nursing homes [1–3]. This model encompasses objective and subjective components, including perceived QoL, the objective environment, psychological well-being, and behavioural competencies. Two types of instruments are employed to evaluate the QoL of nursing home residents: self-assessment, which involves standardised questionnaires administered during an interview with the resident [4–8], and, in instances where the resident cannot respond due to severe impairment, hetero-assessment, entailing the observation and recording of behaviours by family or caregivers [7, 9–11]. However, historical and contemporary research underscores a critical observation: proxies always provide a lower QoL rating than those reported by individuals [12–15]. Nevertheless, extensive research indicates that individuals with mild to moderate impairments can effectively self-assess their QoL [12, 16–19], including those in more advanced stages of impairment [17, 18, 20].

In addition to these methods, semi-structured interviews, guided by the adapted Farquhar methodology [21], present another valuable approach for delving into the nuances of QoL among nursing home residents [21], present another valuable approach for delving into the nuances of QoL among nursing home residents. This qualitative technique enables a deeper exploration of residents’ subjective experiences and perceptions, offering insights complementary to the data collected through standardised questionnaires. Employing semi-structured interviews allows for a more holistic understanding of the factors that contribute to QoL, incorporating the voices and perspectives of the residents themselves.

This qualitative technique enables a deeper exploration of residents’ subjective experiences and perceptions, offering insights complementary to the data collected through standardised questionnaires. Employing semistructured interviews allows for a more holistic understanding of the factors that contribute to the QoL, incorporating the voices and perspectives of the residents themselves.

The QoL in nursing homes of residents with Alzheimer’s disease is multifaceted and depends on various factors. Many studies have extensively examined the factors that determine the QoL in care facilities, and their findings have been summarised in systematic reviews of the literature [17, 22–26]. Understanding the intricacies of Alzheimer’s disease and the social aspects of nursing homes is crucial for comprehending QoL in these settings. Social aspects such as connection, relationships with family and staff, and engagement in social activities significantly shape residents’ overall well-being and QoL [23–25].

However, the subjective QoL of residents with Alzheimer’s in nursing homes is not routinely assessed like other health indicators, despite recommendations to assess Health-related QoL in chronic diseases [27]. Additionally, different cultures and regions vary in how residential care is approached, practised, and perceived [23, 28]. While there has been progress in research within international literature, it is important to conduct tailored studies within local contexts to fully capture the range of factors that influence the QoL of residents in nursing homes due to their unique sociocultural nuances.

In France, the term “EHPAD” (Établissement d’Hébergement pour Personnes Âgées Dépendantes) refers to a nursing home or residential care home for older people who require assistance and care. A recent study has explored the influence of pre-admission factors on QoL and adaptation in EPHAD residents with dementia [29]. This prospective study is important because it provides a better understanding of the predictors of successful adaptation to a retirement home. In addition, this study has highlighted the need for a better understanding of the predictors of QoL for EHPAD residents throughout their stay. In the absence of an instrument assessing QoL in institutions in France, a proxy was developed to compensate for this shortcoming and assess QoL, particularly within cohorts [30].

One year later, in 2021, the Quality of Life in Alzheimer’s Disease Nursing Home version (QoL-AD NH) was adapted and validated [5]. This was the first scale in the French language to measure the QoL of Alzheimer’s disease patients in nursing homes. This study was also the first real validation and psychometric measure of the well-established QoL-AD scale from Logsdon and modified by Edelman et al. [4, 7].

In the existing literature, there is a noticeable gap in research focused on understanding the impact of sociodemographic predictors on QoL-AD NH scores among French nursing home residents, particularly across varying cognitive levels. Furthermore, few studies have taken a qualitative approach to elucidating the determinants of QoL for EHPAD residents at different cognitive levels [31]. This gap is further pronounced by a lack of a theoretical model specifically tailored to assess QoL in Alzheimer’s disease patients within institutional settings.

The purpose of the study was threefold:

1. To measure the impact of sociodemographic predictors on the QoL-AD NH scores among French nursing home residents across different cognitive levels, examine the questionnaire’s overall scores and individual items, and identify differences in residents’ ratings across cognitive groups.
2. To elucidate the QoL determinants for EHPAD residents at different cognitive levels by analysing responses to semi-structured interview questions and comparing the variation of these determinants with cognitive level.
3. To integrate and compare the quantitative (QUAN) and qualitative (QUAL) findings to comprehensively understand the determinants and predictors affecting QoL among French EPHAD residents across various cognitive stages and to compare these findings with Lawton’s theoretical model.

## Methods

### Study design

**Fig. 1:**
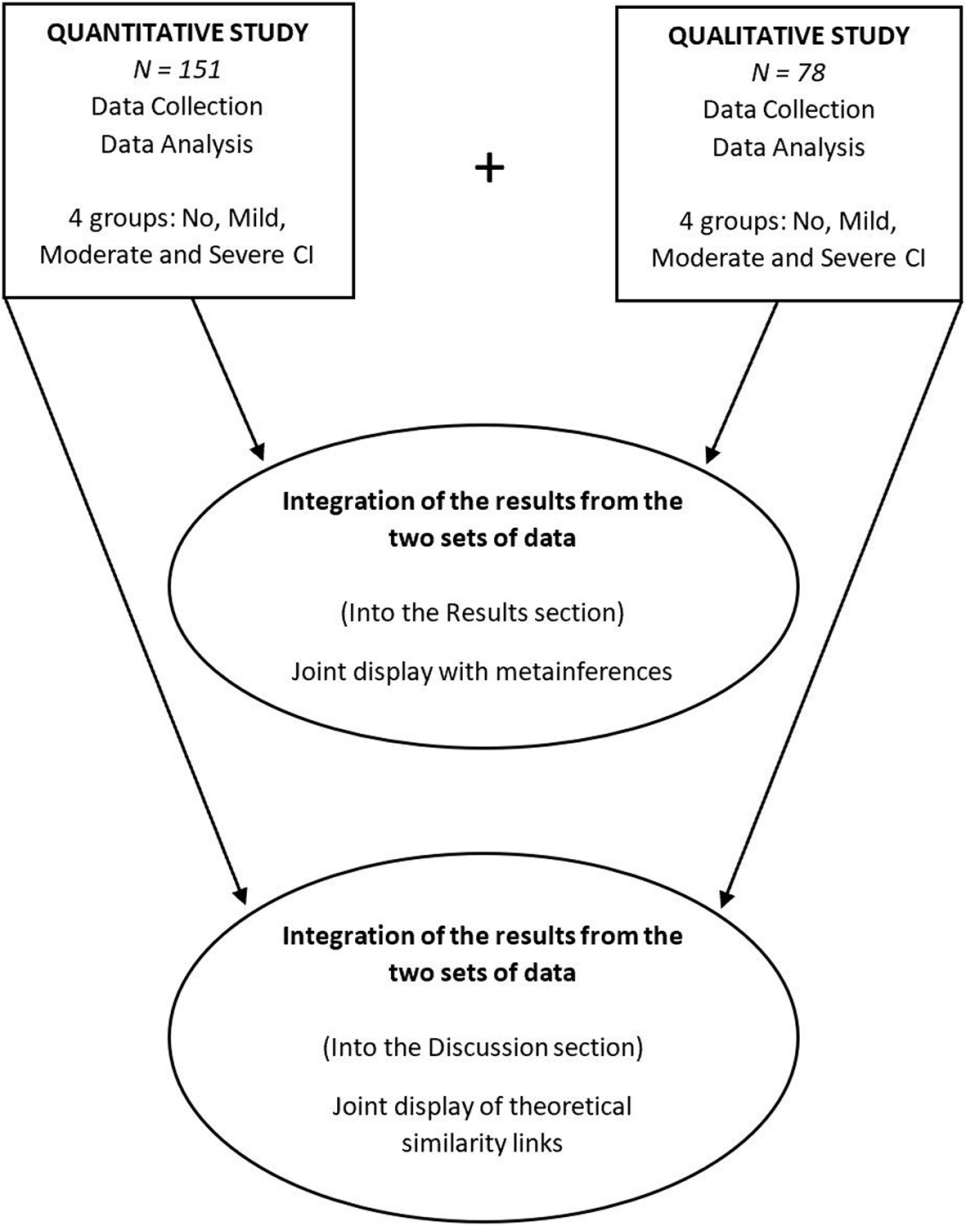
Mixed methods convergent design

Drawing on a pragmatic philosophy, we conducted a mixed-methods field survey using a cross-sectional convergent design [32, 33]. A convergent design necessitates the simultaneous execution of QUAN and QUAL studies during a single phase of the research process. This approach mandates different method sections, independent analyses of each component of the results, and then integration of the results from both studies to formulate conclusions called meta-inferences [34]. We conducted this model without giving precedence to either method. The integrated and mixed analysis of the two datasets is presented in our study’s Results and Discussion sections (Fig. 1).

### Participants

#### Study procedure for resident recruitment

Initially, nursing homes located in the southern Nouvelle-Aquitaine region of France were contacted by phone to seek permission to conduct interviews with the residents of these facilities. After obtaining approval from the nursing home administration, residents and their families were notified about the intention to research the QoL experienced by the residents within the facility. The initial phase of resident recruitment was facilitated by a partnership between the principal investigator (CC) and the nursing home’s healthcare teams. Together, they compiled a preliminary list of residents who, based on their familiarity with the individuals, were considered capable of and likely to consent to participate, aligning with the study’s inclusion and exclusion criteria.

#### QUAN study groups

In this study, we opted for a stratified sample by reasoned choice, primarily due to the specific challenges associated with conducting research in nursing homes. We wanted to create a sample representative of the nursing home population, with residents presenting cognitive impairment at different stages (mild, moderate, and severe) and residents with no cognitive impairment, which we tried to achieve through our inclusion criteria. The sample size for this research was determined through an a priori power analysis using the G*Power software. G*Power was employed to conduct a power analysis specific to general linear models, considering our study’s expected independent and dependent variables. In this analysis, we assumed a statistical power (1—β) of 0.80 and a significance level (α) of 0.05. G*Power suggested a minimum of 150 subjects to achieve statistically significant results.

Inclusion criteria for group formation.

Constitution of a group of residents with no cognitive impairment (NoCI):

- Resident in an EHPAD for at least three months
- Folstein MMS not pathological given sociocultural level

Constitution of three groups of residents with CI at different stages:

- Resident in an EHPAD for at least three months
- Presence of mild cognitive impairment (MildCI): updated MMS of 20-26
- Presence of moderate cognitive impairment (ModerateCI): updated MMS of 10-19

Folstein Mini-Mental State Examinations (MMSE), French version adapted by the GRECO, less than two months old were considered [35, 36]; beyond this period, residents were reassessed by the principal investigator to obtain an updated MMS. In addition, the MMSE scores were adjusted based on each participant’s Niveau Socio-Culturel, or sociodemographic level. This adjustment is crucial as it accounts for variations in educational and cultural backgrounds, ensuring that the cognitive assessment is appropriately tailored to the individual’s context. The sociocultural level helps calibrate the MMSE to provide a more accurate representation of cognitive abilities across diverse groups. For categorisation into four cognitive groups, we adhered to established clinical guidelines that define specific MMSE score ranges for each level of CI [35, 37, 38]. In addition to the usual sociodemographic characteristics, we determined the residents’ level of dependency using the GIR grid. Established by the coordinating doctor, it can range from GIR6 (autonomous) to GIR1 (totally dependent). In our study, we did not calculate the GIR scores directly, which were instead obtained from the residents’ medical records [39].

The exclusion criteria were:

- Residents with significant hearing or vision impairments, even with corrective measures in place.
- Residents who had been hospitalised within the 30 days before the interview.
- Residents with severe cognitive-behavioural issues that would hinder their ability to answer the questions.

#### QUAL study groups

Both the QUAN and QUAL studies employed identical inclusion and exclusion criteria. We constituted a purposive sample of residents of French nursing homes. The residents were divided into four study groups based on their cognitive level as measured by the Folstein MMSE test and their sociocultural level [36]. We formed four groups with as similar as possible sociodemographic variables: a group of residents with NoCI, a group of residents with MildCI, a group of residents with ModerateCI, and a group of residents with SevereCI.

#### Mixed study groups

The two studies, QUAN and QUAL, were not balanced in terms of the number of residents. However, the two samples were linked in the sense that all of the residents in the QUAL study also took part in the QUAN study by answering the study questionnaires.

### Data collection

As part of our research protocol, we opted to begin the with the semi-structured interviews of EHPAD residents, followed by the administration of the standardised questionnaires. This sequence was deliberately chosen in order to minimise the potential priming effects that could arise if participants were first exposed to the closed questions of the questionnaires. By starting with the interviews, we sought to encourage residents to freely express their experiences and perceptions about their QoL, without being influenced by any predefined framework. Although the order of data collection might suggest a sequential approach, our study employed a parallel and convergent mixed methods design as both the qualitative and quantitative studies were conducted independently to completion before their findings were integrated. This order of presentation in our manuscript reflects the simultaneous but distinct nature of the data collection processes, aligning with the principles of mixed methods research.

Data were collected in January and February 2020 before the COVID-19 pandemic.

#### QUAN data collection tools

The administered protocol included the following questionnaires:

The Quality of Life in Alzheimer’s Disease Nursing Home, French version (Qol-AD NH) [5]. The QoL-AD NH is a 15-item questionnaire that measures the QoL of individuals with Alzheimer’s disease residing in nursing homes. These items cover various aspects of life, including physical health, energy, mood, living situation, memory, family, staff, friends, self-image, ability to keep busy, ability to do things for fun, life overall, ability to take care of him/herself, ability to live with others, and ability to make choices in his/her life. The possible answers to the questions were of the Likert type: *poor* (1 point), *average* (2 points), *good* (3 points), and *excellent* (4 points). The scores ranged from 15 to 60. The higher the score, the better the perceived QoL.

The Dementia Quality of Life (DQoL) French version [40].

The DQoL measures the QoL specifically in individuals with dementia. It comprises several domains, including self-esteem (4 items), positive affect/humour (6 items), negative affect (11 items), feelings of belonging (3 items), and sense of aesthetics (5 items). Each item on the DQoL is rated on a 5-point Likert scale, allowing for a nuanced capture of the residents’ perceived QoL. A total score is calculated for each dimension. A high score indicates a better QoL, except in negative affect.

The Geriatric Depression Scale (GDS-15) French version [41].

The GDS-15 is a 15-item screening tool used to identify depression in older adults. It is specifically formatted for elderly populations to minimize the effects of physical illness on the assessment of depression. The GDS-15 asks simple yes/no questions about various symptoms of depression such as “Are you basically satisfied with your life?” or “Have you dropped many of your activities and interests?” Each ‘depressive’ answer is scored as 1 point, thus, the total score ranges from 0 to 15, with higher scores indicating greater depressive symptoms.

#### QUAL data collection tools

We employed a semi-structured interview guide based on Farquhar’s QoL questions [21]. Farquhar is noted for her work in gerontology, specifically developing methods to assess QoL in elderly populations. The five original questions, which we have reworded slightly, were: 1) Could you tell me about your current life? How is your life? (Follow-ups: Why do you say this? What are the reasons why you say this?) 2) What is good about your current life? 3) What is not good about your current life? 4) What would make your life better? 5) What would make your life more difficult? Two questions were added to the original interview guide. The first one “What is important in your current life? What is important to you today?” between the original questions 4 and 5 intends to evaluate what is important for the resident and to test response shift. The second question, positioned at the end, was designed to prompt the resident to discuss their health if they had not addressed it earlier: “Could you tell me about your health (and illnesses if you have them)?”. Using Farquhar’s original version as a starting point, we tested this interview guide with five subjects, reworded it slightly to improve its clarity and retested it with five other subjects to arrive at this final version as presented above.

Residents were interviewed in their rooms, and these interviews were recorded with a dictaphone with their consent. All the residents who answered the questions in the semi-structured interview also answered the QoL-AD NH questionnaire, but not all residents who completed the questionnaire participated in an interview.

### Data analysis

#### QUAN data analysis

First, we addressed missing data through the listwise deletion method, which was consistent with the default settings in SPSS. This approach excluded cases with missing data on any of the variables considered in a specific analysis. The decision to employ listwise deletion was based on its ability to maintain our analyses’ integrity and statistical rigour by ensuring that only complete cases were analysed. This method helps to avoid the potential biases associated with imputation methods or the inclusion of partial data, thus preserving the validity of the analyses conducted. It is important to note that the application of this method impacted the total number of participants analysed in certain statistical tests.

Second, the assumptions of normality and homogeneity of variances were verified using residual graphs. Homoscedasticity was assessed through visual inspection of residual plots. Multicollinearity was assessed using VIF values. A general linear model was used to determine whether sociodemographic variables (age, loss of autonomy, gender, marital status, level of education), depression (GDS-15 score), and cognitive level (no, mild, moderate, severe CI) predicted the total scores for QoL-AD NH. Then, to compare the responses to the 15 items on the QoL-AD NH scale across the four groups, a Kruskal–Wallis test was used. Although the Kruskal–Wallis test identified significant differences among groups, it did not specify where these differences lay. To address this, we conducted pairwise comparisons using the Dunn-Bonferroni post hoc tests to pinpoint the differences between groups. The adjusted p-values were used to better account for Type I errors. We established a significance threshold at p < 0.05 for all inferential tests conducted in this study.

We used IBM SPSS Statistics Release 29 for statistical calculations.

#### QUAL data analysis

The interviews were transcribed using Express Scribe software. In this study, an inductive approach was adopted for the inter-case analysis and coding of the data, allowing the elucidation of themes within and between four groups defined by cognitive level. A variable-oriented strategy was preferred over a case-oriented strategy. This methodological approach aligned with the study’s main objective of identifying QoL predictors as cognitive-level functions. In addition, it is in line with the recommendations of Miles et al. [42]  process for coding data was:

##### Data grouping

The participants were grouped into four categories based on their cognitive levels, setting the stage for both within-group and between-group analyses.

##### Data familiarisation and initial coding by the principal investigator (PI)

The principal investigator (CC) started the analysis by deeply immersing himself in the data, becoming familiar with the content, and noting initial ideas. Following this phase, he performed the initial coding of the data. This process was conducted inductively, allowing codes to emerge organically from the data. This approach ensured that the participants’ perspectives were instrumental in guiding the development of themes.

##### Justification for the choice of themes

During the data analysis process, we did not solely rely on the frequency of occurrences when selecting themes. While frequency can indicate the importance of a theme, it does not always capture the depth or complexity of participants’ experiences. We chose themes that provided an understanding of residents’ experiences and perspectives, even if some of these themes were not mentioned frequently. This approach aligns with the research’s core principle of exploring phenomena in depth and complexity rather than being limited to prevailing trends.

##### Co-author review

Next, the two co-authors (VI & BQ) independently reviewed the initial coding and themes identified by the PI. Their role was to ensure the coherence and robustness of the emerging themes and confirm that they accurately represented the data. Any discrepancies, suggestions for additional themes, or revisions of theme definitions were discussed and resolved in collaboration with the PI.

##### Final consensus and theme validation

All authors convened to review and confirm the emerging themes and finalise the analysis. This collaborative session incorporated feedback from the co-authors’ reviews and integrated insights derived from AI analysis.

Once the primary thematic analysis was complete, we utilized the AI Assist feature in MAXQDA as a supplementary step. This AI tool was deployed to verify the comprehensiveness of our manually derived codes and themes, checking for any potentially overlooked codes or subcodes. Importantly, the AI analysis did not introduce new themes but confirmed the robustness of those identified manually, providing an additional layer of validation.

##### MAXQDA

Analytics Pro 2022 R. 22.7.0 was used as computer-assisted qualitative data analysis software (CAQDAS) to code and analyse the data.

##### Mixed data analysis

Mixed data analysis is presented using joint displays in the Results and Discussion sections. A joint display, also known as an integration display, displays the integration of QUAN and QUAL data in a single table or graph, effectively combining the two types of data [34]. This method makes it easier to compare the outcomes in a clearer, more detailed way. The meaning of these outcomes, when taken together, are known as metainferences.

### Ethical considerations

The study was approved by the Toulouse Ethics Committee (number 2017–064). Information about the study was presented to residents in a way that was adapted to their cognitive impairment stage. For residents with NoCI or only MildCI, written consent was requested after providing clear information in large print. These residents were informed of their right to withdraw at any time without consequence.

A simplified, clearly verbalised form was used for residents with ModerateCI and SevereCI. Their comprehension was regularly checked, and their verbal consent was sought on several occasions: three times before and twice during the study. Permission was also obtained from legal guardians or relatives of residents under legal protection. Consent was checked again if any reluctance was observed during these interactions, and the resident’s right to withdraw was constantly reiterated.

The PI, a psychogerontologist experienced in nursing homes, ensured the well-being of the residents before, during, and after the protocol. Residents were given as much time as they needed to respond, with particular attention given to when they were uncomfortable answering specific questions. Interviews were conducted privately in residents’ rooms to ensure their comfort and confidentiality. Recognising the importance of participant well-being, the protocol was designed to be flexible; if a resident showed signs of fatigue or needed to attend to care routines, sessions could be paused and continued at another time. This approach ensured that the study accommodated participants’ health and comfort needs, allowing for breaks or multiple sessions as necessary.

## Results

### QUAN results

#### Sociodemographic characteristics by groups of the QUAN study participants and descriptive statistics

The study involved 151 participants from seven nursing homes, predominantly women (74.8%). They had an average age of 85.8 years, with varying marital statuses; most were widowed (74.2%). Regarding education, 51% had primary education, while 18.5% had no formal qualifications. Autonomy levels, assessed using the GIR scale, varied with GIR 4 being the most common (33.1%). Cognitive impairment was present at different levels: NoCI (27.8%), MildCI (21.2%), ModerateCI (33.8%), and SevereCI (17.2%). This demographic information is fully presented in Table 1. Descriptive statistics are presented in Additional File 1.

**Table 1:** Sociodemographic characteristics by groups of the quantitative participants (*N* = 151)

	**Total**		**NoCI**		**MildCI**		**ModerateCI**		**SevereCI**	
	**N**	**M/Range/%**	**N**	**M/Range/%**	**N**	**M/Range/%**	**N**	**M/Range/%**	**N**	**M/Range/%**
**Age**	151	85.8/69–100	42	27.8%	32	21.2%	51	33.8%	26	17.2%
60–70	9	6.0%	2	4.8%	3	9.4%	2	3.9%	2	7.7%
71–80	22	14.6%	7	16.7%	5	15.6%	7	13.7%	3	11.5%
81–90	69	45.7%	18	42.9%	15	46.9%	27	52.9%	9	34.6%
91–100	51	33.8%	15	35.7%	9	28.1%	15	29.4%	12	46.2%
**MMSE Mean/Range**		18.00/05–30		26.55/23–30		22.40/19–25		14.77/12–18		8.39/5–10
**Gender**										
Male	38	25.2%	10	23.8%	8	25.0%	14	27.5%	6	23.1%
Female	113	74.8%	32	76.2%	24	75.0%	37	72.5%	20	76.9%
**Marital status**										
Single	22	14.6%	4	9.5%	3	9.4%	8	15.7%	7	26.9%
Married	13	8.6%	2	4.8%	3	9.4%	4	7.8%	4	15.4%
Divorced	4	2.6%	1	2.4%	1	3.1%	1	2.0%	1	3.8%
Widowed	112	74.2%	35	83.3%	25	78.1%	38	74.5%	14	53.8%
**Educational level**										
No qualification	28	18.5%	3	7.1%	4	12.5%	12	23.5%	9	34.6%
Primary education	77	51.0%	22	52.4%	16	50.0%	28	54.9%	11	42.3%
Secondary education	24	15.9%	9	21.4%	8	25.0%	6	11.8%	1	3.8%
Higher education	22	14.6%	8	19.0%	4	12.5%	5	9.8%	5	19.2%
GIR*										
GIR 1	1	0.7%	0	0.0%	0	0.0%	1	2.0%	0	0.0%
GIR 2	39	25.8%	3	7.1%	8	25.0%	19	37.3%	9	34.6%
GIR 3	40	25.8%	4	9.5%	7	21.9%	20	39.2%	9	34.6%
GIR 4	52	33.1%	20	47.6%	13	40.6%	11	21.6%	8	30.8%
GIR 5	15	9.9%	11	26.2%	4	12.5%	0	0.0%	0	0.0%
GIR 6	4	2.6%	4	9.5%	0	0.0%	0	0.0%	0	0.0%
*Note*. *GIR = Level of dependency (from GIR 1, the most dependent to GIR 6, the least dependent)										

#### Handling of missing data

Our analysis initially included 151 participants. The application of listwise deletion due to missing data on relevant variables for our general linear model (GLM) analyses led to the exclusion of 4 cases, representing 2.6% of the total sample. These cases were primarily missing due to incomplete responses in the GDS-15 and DQoL assessments. For correlation analyses, the missing data impacted only 3 cases, resulting in 148 participants being included. This discrepancy is due to the slightly different requirements for complete data in these specific analyses. The minor proportion of data exclusion is unlikely to impact the overall results significantly, maintaining the integrity of our statistical analyses.

#### Results of the general linear model

Conditions of assumptions of normality, homogeneity of variances, and homoscedasticity with a constant spread of residuals across the range of fitted values were satisfied. VIF values were below 1.5.

The model explained 45% of the variance in QoL-AD NH scores (*R*^*2*^ = 0.453, adjusted *R*^2^ = 0.397). Furthermore, the overall model was significant, *F* (13,133) = 8.47, *p* < 0.001, indicating that the chosen predictors significantly explain the observed variability in QoL-AD NH scores. The results presented in Table 2 show that among the predictors, depression had a significant and strong negative impact on QoL-AD NH scores (*β* =  − 3.00, 95% CI: [-3.67, -2.33], *p* < 0.001). The more depressed residents were, the lower their QoL-AD NH scores.

**Table 2:** Results of a general linear model for the sociodemographic factors: loss of autonomy, depression, and cognitive impairment (CI) on QoL-AD NH scores (*N* = 147)

Variable	B	ES	t	Sig.
Age	-.05	.06	-.79	.430
Loss of autonomy (GIR)	.57	.46	1.24	.217
Women vs. men	-.85	1.09	-.78	.438
Marital status				
Single vs. widowed	1.39	1.39	.99	.322
Married vs. widowed	1.05	1.62	.65	.517
Divorced vs. widowed	5.44	2.67	2.03	.096
Level of education				
No qual. vs. High sch. & HE	2.21	1.60	1.39	.168
Primary vs. High sch. & HE	2.28	1.34	1.70	.092
Secondary vs. High sch. & HE	1.73	1.61	1.08	.284
Depression	-3.00	.34	-8.81	.000
Cognitive impairment (CI)				
Severe vs. no CI	-1.18	1.52	-.78	.439
Moderate vs. no CI	-1.43	1.28	-1.12	.267
Mild vs. no CI	-2.76	1.37	-2.02	.046

Additionally, residents with NoCI had significantly higher QoL-AD NH scores (*M* = 38.96, *SD* = 6.25) compared to those with MildCI (*M* = 35.68, *SD* = 6.83; *p* = 0.046, 95% CI for *β*: [-5.47, -0.05]).

Variables such as age, gender, marital status, education, and loss of autonomy did not significantly predict QoL-AD NH scores in this sample.

#### Comparison of responses to the 15 items of the QoL-AD NH scale across the four groups

We performed a Kruskal–Wallis test to determine whether the responses to the 15 items differed from one group to another. The results showed that responses to item 10 “Ability to keep busy daily” differed between the groups, with *KW*(3) = 10.17, *p* = 0.017. The results also showed that the responses to item 12 “Current life in general” also differed between the groups (*KW*(3) = 11.83, *p* = 0.023. We used a pairwise post-hoc Dunn test to reveal the differences. The results showed that residents with MildCI had significantly lower “Ability to keep busy daily” scores than residents with NoCI (*p* = 0.002). Similarly, we found that residents with MildCI also had significantly lower “Current life in general” scores than residents with NoCI (*p* = 0.019).

### QUAL results

#### Sociodemographic characteristics by groups of the QUAL study participants

The study involved 78 participants from seven nursing homes. Participants in the QUAL study had an average age of 87.41, ranging from 69 to 100 years. Participants showed varying MMSE scores regarding cognitive functioning, with an overall mean of 18.41. Those with NoCI had the highest average score (26.68), while participants with SevereCI had the lowest (8.11). The participants’ marital status varied, with the majority being widowed (84.6%). Educational levels varied, with primary education being the most common (52.6%). As assessed using the GIR scale, autonomy levels varied across different groups, with GIR 4 being the most common. This sociodemographic information is fully presented in Table 3.

**Table 3:** Sociodemographic characteristics by groups of the qualitative study participants (*N*  = 78)

	**Total**		**NoCI**		**MildCI**		**ModerateCI**		**SevereCI**	
	**N**	**M/Range/%**	**N**	**M/Range/%**	**N**	**M/Range/%**	**N**	**M/Range/%**	**N**	**M/Range/%**
**Age**	78	87.41/69–100	22	86.45/69–96	20	87.80/74–95	18	87.56/73–100	18	88.00/75–100
60–69	1	1.3%	1	4.5%	0	0.0%	0	0.0%	0	0.0%
70–79	8	10.3%	3	13.6%	2	10.0%	1	5.6%	2	11.1%
80–89	35	44.8%	8	36.4%	10	50.0%	8	44.4%	9	50.0%
90–100	34	43.6%	10	45.5%	8	40.0%	9	50.0%	7	38.9%
**MMSE Mean/Range**		18.41/05–30		26.68/23–30		21.95/19–25		14.67/12–18		8.11/5–10
**Gender**										
Male	14	17.9%	4	18.2%	2	10.0%	3	16.7%	5	27.8%
Female	64	82.1%	18	81.8%	18	90.0%	15	83.3%	13	72.2%
**Marital status**										
Single	7	9.0%	2	9.2%	1	5.0%	3	16.7%	1	5.6%
Married	2	2.6%	1	4.5%	0	0.0%	0	0.0%	1	5.6%
Divorced	3	3.8%	1	4.5%	0	0.0%	1	5.6%	1	5.6%
Widowed	66	84.6%	18	81.8%	19	95.0%	14	77.7%	15	83.2%
**Educational level**										
No qualification	10	12.8%	0	0.0%	2	10.0%	5	27.8%	3	16.7%
Primary education	41	52.6%	10	45.4%	11	55.0%	10	55.5%	10	55.5%
Secondary education	14	17.9%	6	27.3%	5	25.0%	2	11.1%	1	5.6%
Higher education	13	16.7%	6	27.3%	2	10.0%	1	5.6%	4	22.2%
GIR*										
GIR 1										
GIR 2	20	25.6%	3	13.6%	4	20.0%	4	22.2%	9	50.0%
GIR 3	22	28.2%	2	9.1%	5	25.0%	7	38.9%	8	44.4%
GIR 4	26	33.3%	9	40.9%	9	45.0%	7	38.9%	1	5.6%
GIR 5	8	10.3%	6	27.3%	2	10.0%	0	0.0%	0	0.0%
GIR 6	2	2.6%	2	9.1%	0	0.0%	0	0.0%	0	0.0%
Note. *GIR = Level of dependency (from GIR 1, the most dependent to GIR 6, the least dependent)										

#### Within-group comparison

Themes and their subthemes revealing predictors of QoL were extracted for each of the four study groups. We present below examples of quotations supporting the themes.

Group with NoCI.

**Fig. 2:**
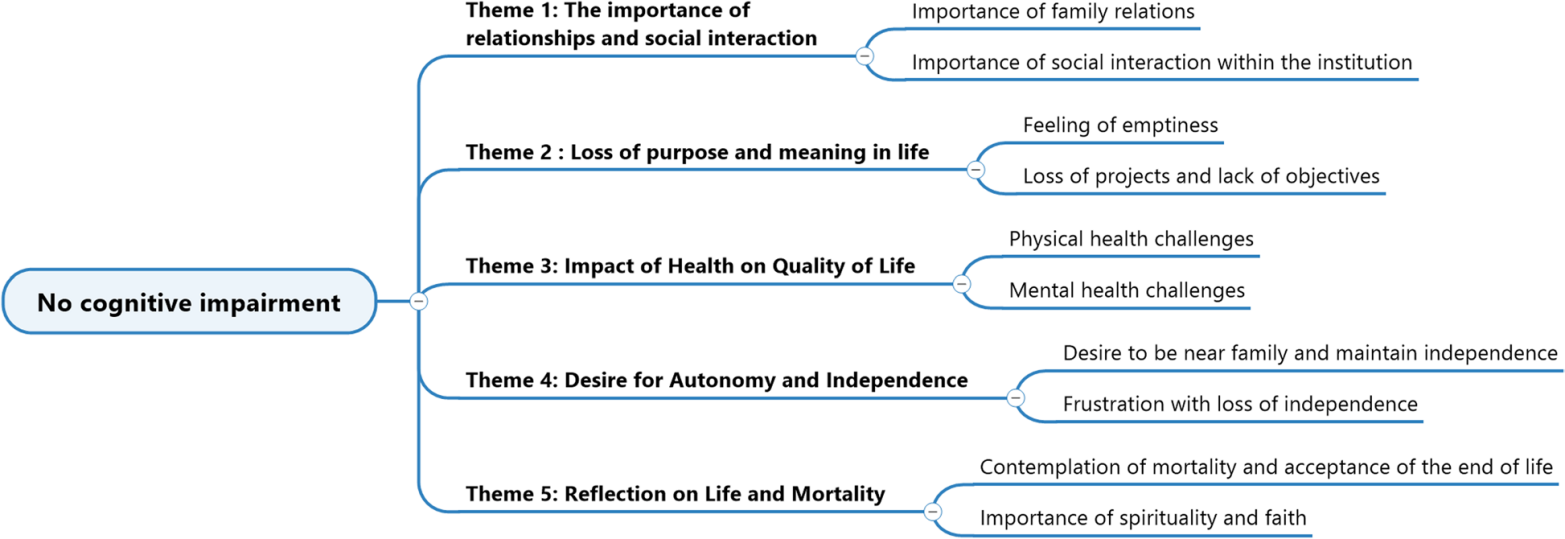
Themes and sub-themes for the group with NoCI

The first group were the residents with NoCI. In Fig. 2, we extracted five themes and two subthemes for each theme.

The significance of social engagement and family ties is key to the perceptions of QoL of residents who do not have cognitive impairment. They wish to sustain fulfilling connections, whether with their loved ones or within the confines of the home. However, they also talk of having no objectives or goals, which makes them feel empty and like their lives have lost value. These residents strive to participate in meaningful activities while maintaining their independence, even if it is relatively institutional, for as long as they can. Their QoL is strongly influenced by health problems, mainly physical ones, and they attach particular importance to maintaining their ability to walk. When it comes to being close to their family or retaining personal independence, the value placed on autonomy is vital, and this sentiment is slightly laced with resentment at their gradual loss of independence. Finally, a recurrent theme is reflection on life and mortality. They reflect on their mortality and appear to be comfortable with death. Although it is not the case for all residents, spirituality and faith play a crucial role in contemplating and accepting mortality.

Example with Mrs. C., 93 years old, NoCI (MMS = 28/30), primary education, widowed, GIR = 4.

(Theme 1).

Interviewer: *What is important in your current life? What is important to you today?*

Mrs. C.: *My children and grandchildren. They come to see me every week, and that makes me very happy. After that, everything else is less important.*

(Theme 2).

Interviewer: *Could you tell me about your current life?*

Mrs. C.: *My life is dull and dreary, and that is all. I lack a reason to be, a reason to occupy myself; and I say to myself, what the hell are you doing on this earth? You have no reason to be.*

Group with MildCI.

**Fig. 3:**
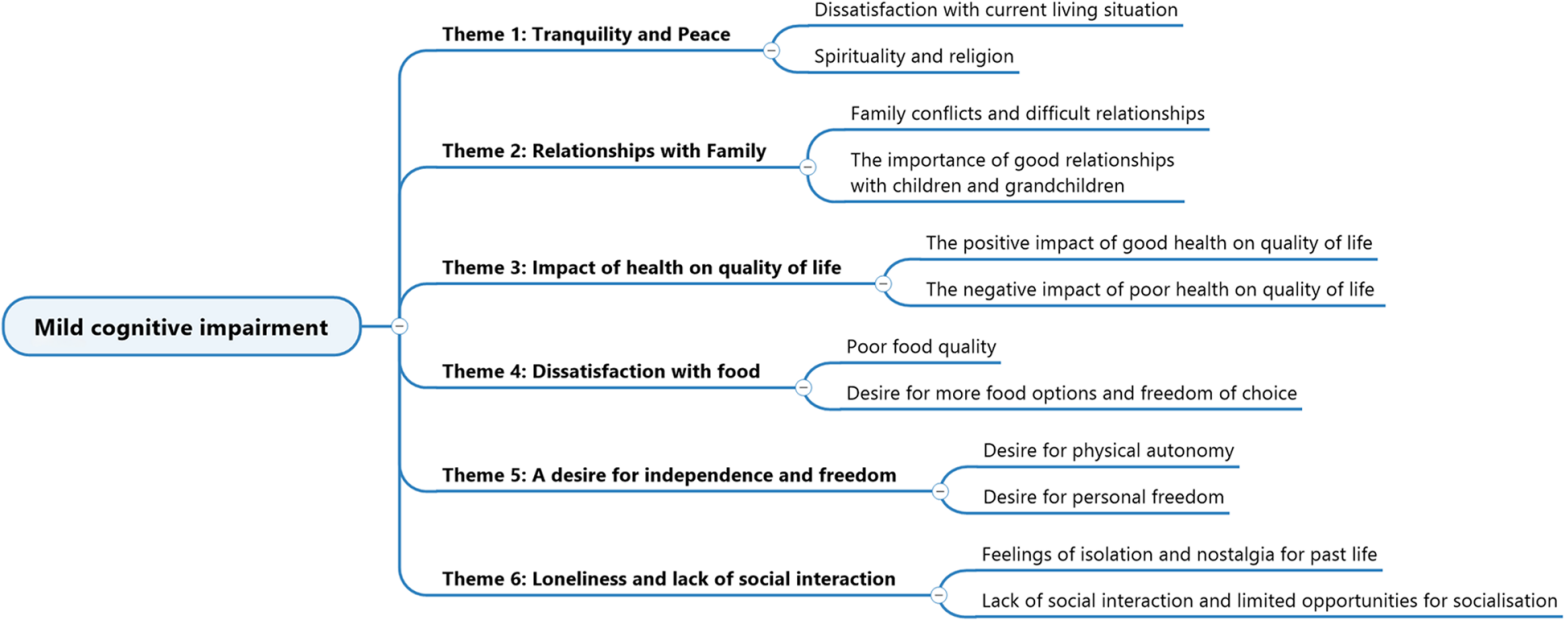
Themes and sub-themes for the group with MildCI

As seen in Fig. 3, we extracted six themes and two subthemes for each theme.

Several themes among the inhabitants with MildCI indicate a general lack of contentment. The subjects fluctuated between enjoying the peace of their surroundings and feeling unsatisfied with their existing living circumstances. Particularly evident is the connection between this unhappiness and the calibre of the food, expressing dissatisfaction with the lack of choice and quality. They also frequently experience feelings of loneliness and nostalgia for their prior lives, characterised by a lack of social connection and participation in activities they criticise, preferring to watch TV and stay in their rooms. Family ties appear significant since disagreements frequently make people feel more unsatisfied. Their physical and mental health is a constant cause of worry, and a common theme is how their health affects their QoL.

Example with Mrs. L., 89 years old, MildCI (MMS = 21/30), secondary education, widowed, GIR = 4.

(Theme 2).

Interviewer: *What is important in your current life? What is important to you today?*

Mrs. L.: *It is when I see my family when they come or take me to their place for a day or to a restaurant.*

(Theme 6).

Interviewer: *Could you tell me about your current life?*

Mrs. L.: *There is nothing to do here; the activities are not worth anything, so I prefer to stay in my room; that way, at least I am quiet, even if I am bored, I got the TV. Moreover, the food is disgusting.*

Group with ModerateCI.

**Fig. 4:**
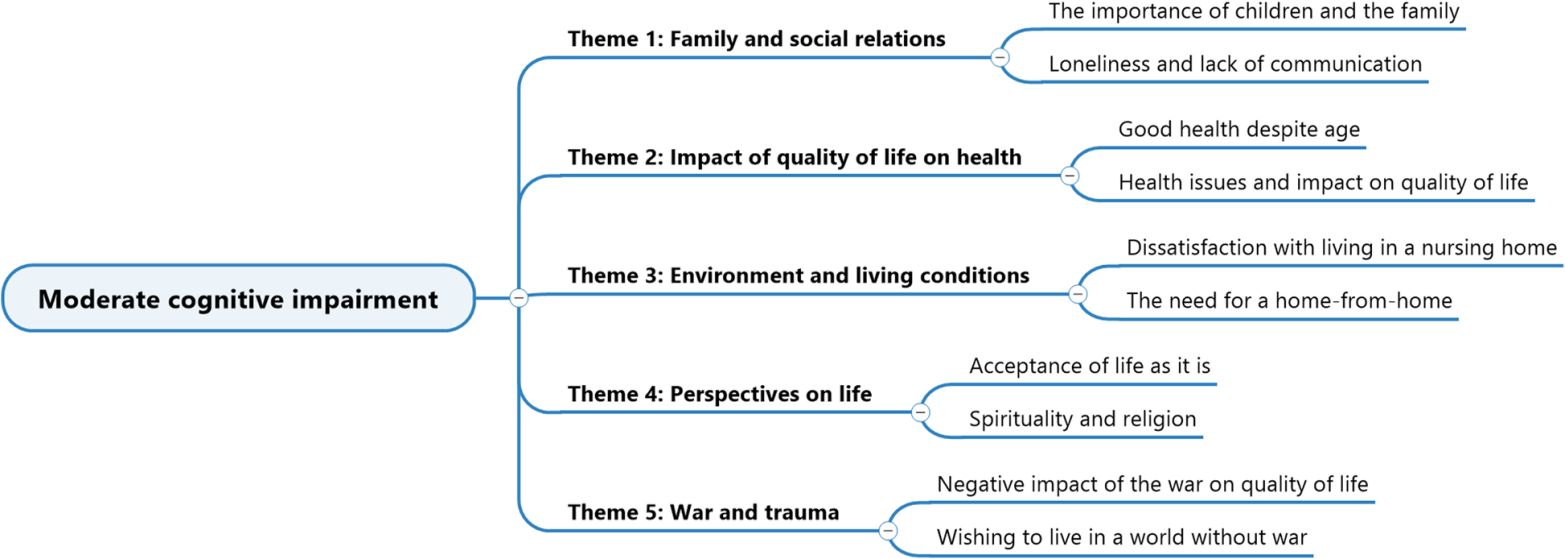
Themes and sub-themes for the group with ModerateCI

As seen in Fig. 4, we extracted five themes and two subthemes for each theme.

The importance of family ties and the desire for a home are heightened among ModerateCI residents. The need to maintain a certain degree of individuality, while residing in a retirement home which frequently fails to meet their comfort and autonomy expectations, is highly valued, as are close relationships with their offspring and relatives. Surprisingly, wartime memories continue to impact their contentment, revealing underlying psychological wounds. Despite their advanced age, some individuals reported outstanding health, whereas others described health issues that substantially impacted their QoL. Lastly, some residents recognised religion and spirituality as a significant, although not dominant, aspect of their lives, which brings them comfort and helps them make sense of their current circumstances.

Example with Mrs. V., 91 years old, ModerateCI (MMS = 16/30), primary education, widowed, GIR = 4.

(Theme 1).

Interviewer: *What is important in your current life? What is important to you today?*

Mrs. V.: *Uh, my family, and especially my son. Ah yes! That is all!*

(Theme 5).

Interviewer: *what’s not going well in your current life?*

Mrs. V.: *I often have flashes where I see the attempted attacks again, and it always scares me to death because I’m afraid they’ll happen again.*

Group with SevereCI.

**Fig. 5:**
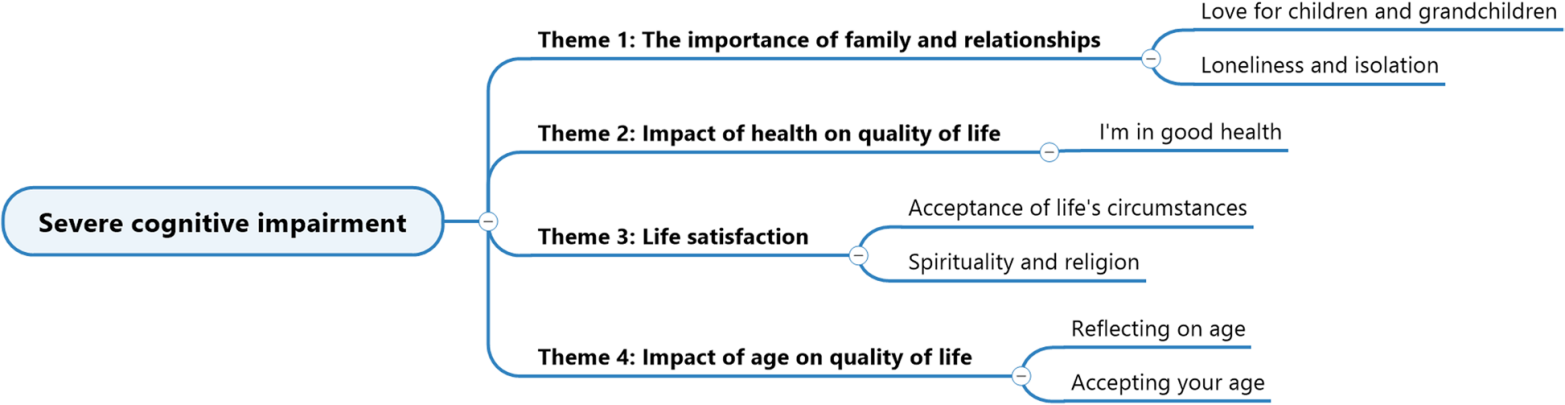
Themes and sub-themes for the group with SevereCI

Before presenting the findings regarding the group of residents with SevereCI, it is important to acknowledge the limitations of our analysis. Given their condition, only two out of the eighteen members could express their QoL. Therefore, the themes we have identified are based on the perspectives and experiences of these two individuals, along with a few others, which may not fully represent the sentiments of the whole group because we did not reach saturation for this group. As a result, the themes for this group were not as rich as those of other groups. Although these two individuals could communicate their QoL, it is worth noting that they might still face challenges in conveying their experiences and emotions. As a result, we may have missed subtleties and nuances during our analysis. Despite these limitations, our analysis provides insights and is a foundation for further research and discussions. As seen in Fig. 5, we extracted four themes and two subthemes.

Family ties remain important to individuals with SevereCI. Despite their often brief and disorganised speech, they express undying love for their children and grandchildren. However, they also seem to be feeling a little lonely and alone, and experience a desire and longing for “home.”. Their health is a minor worry, and they are happy that they are healthy despite their advanced age. Their acceptance of life’s circumstances is influenced by spirituality and religion, although these issues are not always front of mind for them. Their testimonies also contain scattered reflections about ageing and accepting their age.

Example with Mrs. R., 87 years old, SevereCI (MMS = 08/30), secondary education, single, GIR = 4.

(Theme 1).

Interviewer: *What is important in your current life? What is important to you today?*

Mrs. R.: *Oula, euh… family, yes, yes!*

(Theme 3).

Interviewer: *Could you tell me about your current life?*

Mrs. R.: *I accept my circumstances. I am at the end.*

#### Inter-group comparison

Depending on their cognitive level, cross-case analysis between the various study groups revealed several similarities and differences in the factors influencing residents’ QoL.

Similarities:

*The importance of relationships with family:* There was unanimity when evaluating what was important for the residents of each group. Relationships, particularly with the family and especially children and grandchildren, were the most important thing for them now, for all resident groups.

*Social engagement within the institution*: All the groups made little mention of everyday activities, including entertainment activities in the nursing home.

*The impact of health on QoL*: All groups know that their health, whether excellent or poor, directly influences their current QoL. When health was not mentioned and they were asked whether it was important to their QoL, almost all residents replied that it had a major impact on their QoL. When asked why they had not mentioned their health when asked what was important to them, the majority replied that they had not thought about it or that it was not interesting to the interviewer.

*Religion and spirituality*: Although not the most essential factor, all groups cited aspects of their religion or spirituality as influencing their perception of QoL.

An internal mixed analysis using MaxQDA to compare primary sociodemographic data between the four groups revealed no obvious differences in their perception of QoL.

Differences:

*Need for independence and freedom*: This characteristic was especially prominent among residents with NoCI and MildCI and was mentioned far less often by residents with ModerateCI and SevereCI.

*Social engagement within the institution*: Residents with CI, particularly those with ModerateCI and SevereCI, value social interaction less than residents with NoCI. Residents with MildCI were the least engaged in activities and complained of a lack of interesting activities within the institution, preferring to watch TV.

*Perspective on life and age:* Residents with MildCI showed unhappiness, while those with NoCI expressed annoyance at losing independence. In contrast, residents with ModerateCI and SevereCI appeared more disposed to accepting their age and living conditions.

*Health on QoL:* Concerning health, the residents with NoCI and MildCI talked more about their health than other groups and mainly raised the issue of losing physical and motor autonomy, particularly the ability to walk.

In summary, the inter-group comparison highlighted both similarities and differences in the factors influencing residents’ QoL. Table 4 provides a comprehensive overview of the key themes impacting QoL for each group in which can be found a detailed breakdown of these themes, including their relative importance across different cognitive impairment groups.

**Table 4:**
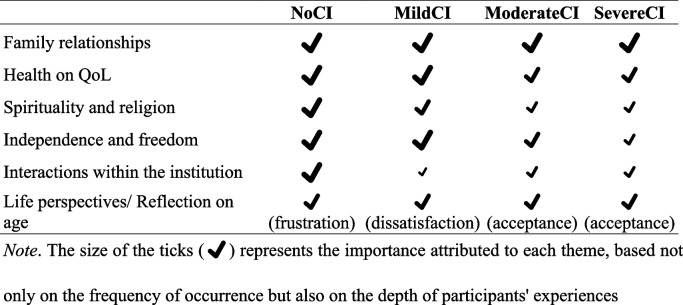
Comparison of key themes impacting qol across different cognitive impairment groups

The findings from the QUAL study distinctly highlight that certain factors including family relationships, health, spiritual beliefs, autonomy, and interactions in the institution hold significance for all residents, regardless of their cognitive state. The consistent relevance and communal nature of these factors suggest that, even though cognitive challenges might modify some perceptions or encounters, these key areas are universally recognised as vital to the perceived QoL of every resident.

**Fig. 6:**
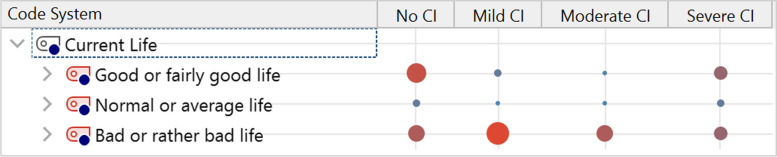
Current Perceived qol across the four groups of residents

Beyond the extracted themes, the results showed that most residents reported that their lives were poor or fairly poor, except for residents with NoCI who were the most likely to feel they had a good QoL (Fig. 6). Residents with ModerateCI, and especially those with MildCI, were overwhelmingly of the opinion that their QoL was poor or fairly poor.

### Mixed results

As illustrated in Table 5, the mixed results are presented through a joint display.

**Table 5:** Joint display of quan and qual findings on predictors of qol in four groups of nursing home residents

Predictors	QUAN study	QUAL study	Meta-inferences
**Sociodemographic** **Data and QoL**	The sociodemographic data did not significantly predict QoL-AD NH scores, including between residents with NoCI and residents in the other three groups with CI at different levels.	There were no apparent differences between the four groups of residents when their perceptions of QoL were compared using an analysis that included sociodemographic information.	*Convergence* Both studies agree that sociodemographic data do not influence the QoL of nursing home residents.
**Depression and QoL**	GDS15 depression scores significantly predict QoL-AD NH total QoL score (*β* = -3.00, *p* = .000). Residents with MildCI had the highest depression scores (5.32 + 3.73).	The themes ranging from tranquillity and peace to often difficult family relationships, dissatisfaction, loneliness, uncertain health, and lack of control highlight various facets of the depressive experience, whatever the group, more marked among residents with MildCI.	*Convergence* The convergence between QUAN and QUAL results is demonstrated by the significant influence of depression scores on QoL, which is corroborated by several emerging themes in the QUAL analysis. This is especially true for MildCI
**Perceived QoL**	A significant difference in the total score (*β* = -2.76, *p* = .046) and the general Qol-AD NH “Current life in general” item score [*U*(1) = -22.33, *p* = .021] between residents with no CI and those with MildCI, with the latter having a poorer perceived QoL on both scores.	Residents with MildCI were the most likely to have a poor QoL in the nursing home, clearly expressing dissatisfaction and poor QoL compared with residents in other groups.	*Convergence* Both studies highlight that residents with MildCI have a poorer perception of their QoL than those with NoCI.
**Daily occupation and QoL**	Residents with MildCI had significantly lower perceived “Ability to keep busy daily” scores on the QoL-AD NH scale (2.55 + .75) than residents with NoCI (2.03 + .71).	Residents with MildCI complained most about a lack of activities and occupations within the institution, attributing the lack of occupations to external causes (not enough activities, criticism of them…). They did not leave their rooms often and watched TV.	*Expansion* The QUAN and QUAL results expand on each other to give a more complete view of the situation. Although residents with MildCI perceive themselves as having a reduced ability to occupy themselves daily, the QUAL data add a layer of understanding by showing that these residents attribute their lack of occupation to external causes, such as the lack of activities offered by the institution. Thus, they may feel both personally unable to occupy themselves and dissatisfied with the activities on offer, reflecting a complexity of lived experience that would not be fully captured by a single research method.

Firstly, the results of the two studies showed that QoL was not influenced by sociodemographic data (age, gender, marital status, level of education, level of dependency). The studies also converged on the theme of depression. Depression was a significant predictor of QoL within both the general linear model and the QUAL study, which revealed several emerging themes related to residents’ depressive aspects. Moreover, the combined results revealed that the residents with MildCI had a poorer perception of their QoL than those with NoCI. Finally, we found results that, at first glance, may seem contradictory but illustrate an expansion of our understanding of the phenomenon under investigation. When answering the questionnaire, these residents attributed their ability to keep busy during the day to internal factors. Yet when discussing their daily activities, they attributed their level of occupation to external factors, such as the lack of engaging activities offered by the institutions. This suggests that they may feel both personally unable to keep busy during the day and dissatisfied with the opportunities offered by the institution. This dual finding reveals a complexity of lived experience that would not have been fully captured if only one research method had been used.

For all groups, we also compared the side-by-side results to QoL-AD NH, and compared each item to the free discourse on their QoL. Although there were convergences, the two sets of data were disconnected, providing different information about their QoL.

## Discussion

### QUAN results discussion

First, sociodemographic data did not predict QoL-AD NH scores, aligning with previous studies [23]. Second, the results from the general linear model showed that depression significantly predicted QoL-AD NH scores. Notably, residents with MildCI displayed the lowest QoL scores and exhibited slightly higher levels of depression compared to those with ModerateCI. This finding highlights depression’s profound influence on QoL, supported by extensive systematic literature reviews [17, 22, 23, 43, 44].

Additionally, a significant difference was observed in QoL scores between residents with MildCI and those with NoCI, with the latter reporting higher QoL. This suggests that even MildCI can significantly impact reported QoL, echoing recent research that underscores the broader effects of CI on QoL [17, 45]. Symptoms such as depression, stigma, and hopelessness, commonly experienced by individuals in the early stages of Alzheimer’s disease, further diminish their QoL.

Further analysis revealed that early stages of CI can markedly affect daily activities and engagement. These findings suggest that targeted interventions could significantly enhance QoL for residents with MildCI. Moreover, the sensitivity of the QoL-AD NH scale in distinguishing between different cognitive states, evident in both total and item scores, suggests good discriminant validity. This initial evidence of the scale’s sensitivity needs further validation in studies testing its responsiveness to change over time.

### QUAL results discussion

This QUAL study aimed to explore the determinants of QoL perceived by nursing home residents according to their level of cognitive impairment. Our results highlight several similarities and differences between the four groups studied.

First, family relationships are a key determinant of QoL across all cognitive groups. Our findings align with the Harvard Study of Adult Development, which emphasises the quality of relationships to overall well-being and happiness [46]. Despite differing contexts—ours in nursing homes and Harvard’s in a broader adult population—the value of meaningful relationships is a consistent theme. These findings echo systematic reviews that identify family connections as a crucial predictor of QoL [23–25].

The similarities between the importance of family relationships and health indicate that some needs and values go beyond cognitive impairment levels. This suggests that any future actions or plans in EHPADs should consider these aspects as key to enhancing the QoL for residents.

Spirituality and religion were common themes for all groups, though less so for ModerateCI and SevereCI. Indeed, it would seem that these results align with previous studies, namely that spirituality and religion remain important elements of QoL even for residents with Alzheimer’s disease [16, 26, 47]. On the other hand, these results and those cited above call into question a recent systematic review of QoL instruments which recommended excluding assessing spirituality and religion for those with Alzheimer’s disease, only recommending it for those with NoCI [48].

Engagement within the facility highlights the unique challenges specific groups face based on their cognitive condition. For instance, individuals with MildCI may have particular or inadequately addressed activity requirements. This point will be addressed in the mixed results discussion.

MildCI residents had strong complaints about the food offered at their institution. Indeed, good meals with quality food and the opportunity to make their own choices would improve residents’ QoL [49].

In our study, ModerateCI and SevereCI residents were more willing to accept their age and living conditions, a point which has not been as strongly emphasised in the existing literature. In contrast to these two groups, NoCI residents were frustrated with their ageing condition and loss of autonomy, especially walking, while MildCI residents were dissatisfied and complained about their condition.

In summary, these similarities and differences demonstrate the necessity for customised care that caters to the needs of each resident group while considering factors that contribute to a high QoL for all.

To complete the discussion of the results, they also showed that the vast majority of residents said that their lives were bad or rather bad. This contradicts the findings of Conversat-Nigay et al. [50], who showed that residents surveyed in their study felt that they had a very respectable QoL, with the vast majority expressing satisfaction.

As with our study, Byrne et al. [51] has shown that residents talked little about their health despite its importance. This can partly be explained by a lack of awareness of disorders, likely affecting the residents with moderate and severe CI, but also by the response shift phenomenon described by Sprangers and Schwartz [52]. This adaptive phenomenon is linked to coping and makes it possible to maintain QoL despite physical or mental deterioration. Our study’s population of nursing home residents was very advanced in years and had high levels of dependency linked to physical and motor disabilities. However, while health appeared to be an important factor in residents’ QoL, it was not perceived as essential since several residents in very poor health felt they had a good QoL.

In conclusion, our QUAL research mainly highlighted the fragility of residents with MildCI. They face particular difficulties adapting to life in an institution and experience more complaints and depression than other residents. We will come back to this in the next section.

### Mixed results discussion

Residents in all groups rated their QoL more negatively orally than they did using the QoL-AD NH questionnaire. The results also showed that, save for very few residents, those with SevereCI were ultimately more able answering the closed questions in the QoL-AD NH than the open questions in the semi-structured interview guide.

Our side-by-side comparison of the residents’ QoL-AD NH and discourse on their QoL found that the two sets of data were disconnected and each one revealed different information about QoL. This reflects the observations of an older study which compared QoL between QUAN and QUAL results [52].

As already stated, sociodemographic variables did not predict QoL-AD NH scores; this was also the case in the QUAL analysis, where a mixed analysis using MaxQDA integrating sociodemographic variables did not reveal any differences in residents’ perceived QoL.

In the QUAL study, we observed that residents with MildCI frequently reported their current life as bad, whereas most residents with NoCI described their life more positively. Consequently, the results of our two studies converge on the fact that within our sample, residents with NoCI reported a better perception of their QoL than residents with MildCI.

The QUAN study also showed a significant difference in response to a QoL-AD NH item relating to the “Ability to keep busy daily.” Residents with MildCI scored significantly lower than those with NoCI. In addition, the QUAL study revealed that residents with MildCI complained most about a lack of activities within the nursing home, attributing the lack of occupation to external causes. These results may seem contradictory for residents with MildCI because, in the QoL-AD NH questionnaire, these residents also gave a negative assessment of their own ability to keep busy during the day (internal attribution). To explain this difference between the two groups, we need to refer to the QUAL study. The latter shows that the residents surveyed with MildCI preferred to stay in their rooms, particularly to watch TV, whereas the residents with NoCI watched TV but also played in groups and took part in the activities offered by the nursing staff. Faure and Osiurak [53] have shown that residents who invested more in collective spaces had a better QoL than those who remained in their private spaces. Other studies have shown that residents involved in social activities had a better QoL [54–56]. However, one study has shown that nursing home residents were largely inactive during the day [57]. Furthermore, in addition to involvement in social activities, activities of daily living, including in residents’ bedrooms, can impact QoL, as some studies have shown [58].

Our study aligns with the literature, which shows the importance of social engagement for QoL, but adds an important nuance concerning variations according to cognitive level [23, 24, 59]. In light of these findings, revisiting the psychosocial aspects affecting residents’ QoL when considering their engagement in daily activities is crucial. As previously discussed, our findings are consistent with the study by Villarejo-Galende et al. [45], which shows a negative correlation between cognitive impairment and QoL, largely attributed to factors like depression and stigma. These emotional and psychological states may be barriers to engaging in more social activities or spending time in communal areas, thus reducing overall QoL. Moreover, there are probably intermediary processes linked to perceived stress and perceived control, as well as feelings of competence, self-efficacy, and self-esteem that enable residents to rationalise their avoidance behaviour [60]. This may have a protective effect on them. In addition, it is possible to view this as unmet or misunderstood needs, as described in the theory of unmet needs in dementia [61–63].

These emotional and psychological states influence residents’ involvement in social activities and raise important theoretical questions about how we understand and measure QoL in this context. This sets the stage for a deeper examination, using joint displays, to explore the complexities of QoL in nursing homes from a comparative and theoretical standpoint.

#### Joint displays: comparative analysis and theoretical implications

We present two joint displays to shed light on QoL in nursing homes: one comparing QUAL themes to the QoL-AD NH and DQoL scales, and the other evaluating these dimensions using Lawton’s model.

**Fig. 7:**
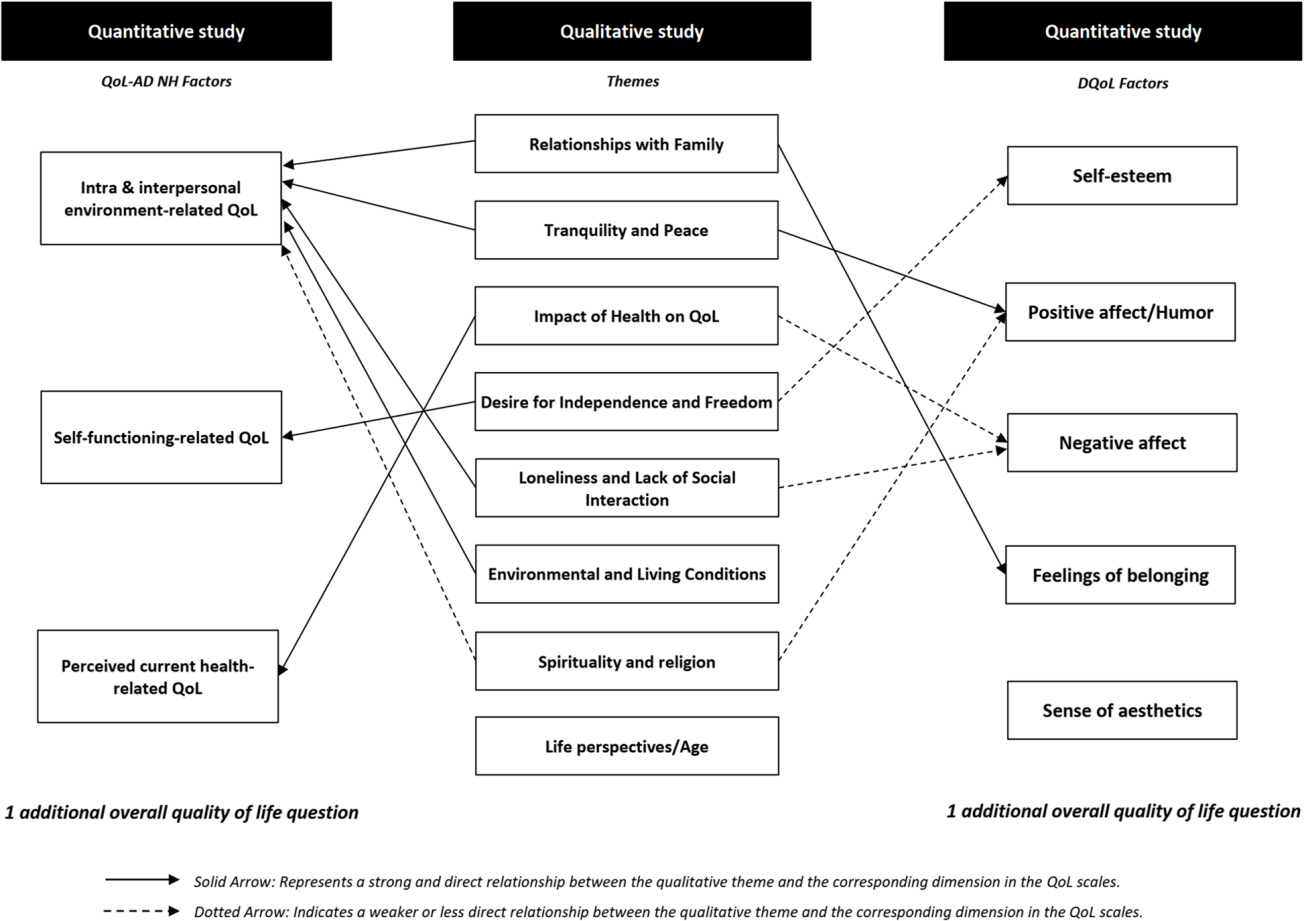
Joint display of relationship between qual themes and dimensions from the QUAN Scales (QoL-AD NH & DQoL)

The first relates the QUAL analysis themes to the dimensions of the QoL scales, QoL-AD NH and DQoL (see Fig. 7).

In this joint display, the strongest relationships are indicated by solid arrows, while dotted arrows represent less obvious links. It also shows the relationship between the themes identified in the QUAL analysis and the different dimensions of the two QUAN scales. Themes such as Desire for independence and freedom” resonate strongly with the “Personal functioning” dimension of the QoL-AD NH, whereas “Family and social relationships” corresponds more closely to the “Intra- and interpersonal environment” dimension of the same tool. Concerning the DQoL, although certain themes, such as “Impact of health on QoL” can be linked to dimensions such as “Negative emotions” the link seems less obvious and less frequent. Its current formulation suggests that the DQoL may not capture some crucial aspects of the resident’s QoL that the QoL-AD NH can identify.

To conclude, this graphical representation reinforces our finding that the QoL-AD NH provides a more comprehensive and nuanced measure of the QoL in this context. These results reflect the appropriateness of tools for measuring QoL in nursing homes and suggest that improvements or adaptations may be needed to capture the complexity and diversity of residents’ experiences. In addition, these results call for a plurality of measures, with at least one QUAN scale and one QUAL interview, to capture elements that may not have been captured with the QUAN instrument.

We also wanted to return to Lawton’s theoretical model assessing QoL in Alzheimer’s disease patients. In Table 6, we present a joint display of Lawton’s sectors and their correspondence with QoL-AD NH factors and themes from the QUAL study.

**Table 6:** Joint display of Lawton’s sectors and their correspondence with QoL-AD NH factors and themes from the QUAL study

QoL-AD NH factors or items	Lawton’s sectors	Themes from the QUAL study
Item N°3: Mood Item N°9: Self image Item N°13: Ability to take care of oneself	**Psychological well-being**	Tranquility and peace Perspectives on life War and trauma Loss of purpose and meaning in life Reflection on life and mortality Feelings of isolation and nostalgia for past life Spirituality and religion
Factor: Intra & interpersonal environment-related QoL	**Objective environment**	Relationships with Family Importance of social interaction within the institution Environment and living conditions
Factor: Self-functioning-related QoL and “Perceived current health-related QoL”	**Behavioural competence**	Impact of health on QoL Impact of age on QoL Lack of social interaction and limited opportunities for socialisation
Total score QoL-AD NH General item N°12: Life in general	**Perceived QoL**	Desire for Autonomy and Independence Life satisfaction Spirituality and religion

The joint display allows us to make some interesting observations about the relationships between the dimensions of the QoL-AD NH and the QUAL themes in the sectors of Lawton’s theoretical model. The first obvious observation is that the QUAL themes are largely aligned with the dimensions of Lawton’s model, thus confirming the usefulness of this theoretical model in assessing the QoL of residents in nursing homes.

In our study, we placed “Spirituality and Religion” at the nexus of psychological well-being and perceived QoL, acknowledging their integral role in human experience. These dimensions offer frameworks for emotional regulation and shape daily perceptions and interactions, directly influencing perceived QoL. This highlights the pivotal role of spirituality and religion in shaping overall QoL.

However, it is clear from the table that the QoL-AD NH appears to have shortcomings, particularly in measuring psychological well-being. While the QUAL data provide a clear picture of psychological well-being, including themes such as tranquillity, reflection on life, and mortality, the QoL-AD NH seems to offer a much more restricted perspective with its “Mood,” “Self-image,” and “Ability to take care of oneself” items. Instead these are only individual items and do not belong to a “Psychological well-being” dimension that did not emerge in the factor analysis used to validate the tool [5]. This suggests that the QoL-AD NH may not be sufficiently sensitive to the complexities of psychological well-being. This lack of sensitivity is worrying because it could lead to underestimating older people’s emotional and existential needs, essential components of their overall QoL. It also highlights the need to rethink or supplement standardised scales such as the QoL-AD NH to better capture the full range of emotional and psychological experiences of older people in institutions.

In conclusion, our mixed analysis, which draws on Lawton’s model, emphasises the significance of adopting more thorough methodological and theoretical approaches for a more precise and nuanced assessment of the QoL of nursing home residents.

Globally, our mixed methods study comprehensively understood QoL among older adults in care facilities, using three joint displays for nuanced interpretation. The first display revealed gaps in evaluations of daily activities and self-occupation. The second showed that the QoL-AD NH aligned more closely with QUAL findings than the DQoL, suggesting its potential to capture subtleties in residents’ experiences even if it lacks some elements. The third incorporated Lawton’s model, highlighting the QoL-AD NH’s limitations in measuring psychological well-being. This multifaceted approach offers cross-validated, enriched insights, affirming its relevance for future research in this complex field.

### Limitations

This study has some limitations that should be noted. The findings may lack generalisability due to the non-probability sample, confinement to regular nursing homes, and the specific French context. The cognitive impairment classification relies solely on MMSE scores, not considering other instruments such as the Clinical Dementia Rating (CDR). We also faced unequal sample sizes across groups and did not reach data saturation for the SevereCI group, affecting statistical power and representativeness. Additionally, the study’s cross-sectional design limits its temporal scope. Self-assessed QoL measures are subject to residents’ cognitive and communicative abilities. Lastly, the study concluded just before the COVID-19 pandemic, which could also impact generalisability.

### Implications

#### For clinical practice in nursing homes

Personalised, Multidimensional Assessment: The aim is to propose provisional recommendations to professionals for a personalised, multidimensional assessment of residents’ QoL in France’s nursing homes. These interim recommendations will then be followed by a more rigorous approach based on the recommendations for good professional practice in the social and medico-social sectors issued by France’s Haute Autorité de Santé [64].

Vulnerable Residents: The results show the importance of looking after the most vulnerable residents, particularly those with MildCI, and offering them personalised and meaningful activity plans.

Family-Centred Approach: The results also call for much greater attention to be paid to families in EHPADs and for a family-centred approach to be adopted in addition to the resident-centred approach, which must be paramount.

#### For public policy

Detecting and Managing Depression: Public authorities could promote recommendations for better detection and management of depression in French nursing homes.

Financial Incentives: The development of social and therapeutic activities within nursing homes could be encouraged financially, focusing particularly on the frailest residents.

Awareness Campaigns and Therapeutic Programs: Advocate for public awareness campaigns to emphasise the importance of family ties in enhancing the well-being of nursing home residents and support the implementation of therapeutic programs for family caregivers to encourage greater participation in care.

### Directions for future research

In future research, it would be worthwhile to have an integrated model of QoL in Alzheimer’s disease in nursing homes, as there is no specific model to date that is theoretically sound, apart from the few determinants that have been revealed. In addition, future studies should evaluate hetero-assessment of QoL as a complement to self-assessment. Conducting further research in France on the daily activities of residents in EHPADs and their involvement in these activities while respecting their wishes and preferences would be worthwhile. Finally, it would be important to research residents’ QoL in protected environments, such as Alzheimer’s insulated and reinforced accommodation units (UHR).

## Conclusions

In this research, we conducted a study using mixed methods to explore the factors that influence the QoL of individuals living in nursing homes in France. We specifically examined how cognitive impairment impacts their perception of their well-being. The QUAN results revealed that depression and MildCI played a role in determining self-reported QoL. Furthermore, we found that residents with MildCI had a more negative view of their overall QoL and poorer ability to stay engaged daily than those with NoCI. The QUAL study highlighted several common determinants across all groups, such as the importance of family relationships and health. However, it also revealed differences between the groups. Notably, residents with MildCI complained more about a lack of activities and poor adaptation to institutional life.

The mixed analysis allowed for a deeper investigation of these results, demonstrating that complaints about daily inoccupation could reflect personal difficulties and a lack of activities offered by the institution. Furthermore, a comparison with Lawton’s theoretical model highlighted the QoL-AD’s limitations for assessing psychological well-being.

In conclusion, this research shows the complexity of QoL in French nursing homes and the need for a mixed approach to understand this multidimensional phenomenon better. It highlights certain vulnerable groups and areas for practice and policy improvement, such as better screening for depression or developing tailored social activities. The findings prompt a rethinking of assessment tools to better capture the subtleties of residents’ experiences. They also underline the value of combining QUAN and QUAL approaches to understand this complex research topic better.

### Supplementary Information

Supplementary Material 1.

## Data Availability

QoL-AD NH is distributed by Mapi Research Trust on behalf of the copyright owner (Rebecca Logsdon): © 1996, Rebecca Logsdon, PhD; University of Washington. QoL-AD NH contact information, provision, and permission to use: Mapi Research Trust, Lyon, France, https://eprovide.mapi-trust.org. Farquhar’s adapted interview guide is available from the corresponding author. The datasets generated and/or analysed during the current study are not publicly available due to privacy and ethical restrictions related to the sensitive data collected from nursing home residents. However, they are available from the corresponding author upon reasonable request, with appropriate ethical safeguards.

## Abbreviations

ANESM: National agency for the evaluation and quality of social and medico-social establishments and services
CERNI: Non-interventional Research Ethics Committee
CI: Cognitive Impairment
DQoL: Dementia Quality of Life
EHPAD: Établissement d’Hébergement pour Personnes Agées Dépendantes
GDS-15: Geriatric Depression Scale 15 items
GRECO: Reflection Group on Cognitive Assessments
MildCI: Mild Cognitive Impairment
QoL: Quality of Life
ModerateCI: Moderate Cognitive Impairment
NoCI: No Cognitive Impairment
PI: Principal Investigator
QoL-AD NH: Quality of Life in Alzheimer’s Disease Nursing Home version
SevereCI: Severe Cognitive Impairment
