# Supplementary material for: Determinants of quality of life in French nursing home residents across cognitive levels: a comparative study using convergent mixed-methods
Source: BMC Geriatr. 2024 Jul 30;24:636. doi: 10.1186/s12877-024-05226-4 (PMC11290140; doi:10.1186/s12877-024-05226-4)
Supplement: Supplementary file 1 — Supplementary Material 1. [file 12877_2024_5226_MOESM1_ESM.pdf]

Title of data: Descriptive statistics of the quantitative study

Description of data: Descriptive statistics for continuous variables in the study; Descriptive statistics for scores on QOL-AD NH items; Pearson correlations between study variables (*N* = 148); Cronbach's alpha coefficients for study variables

Table S1

Descriptive statistics for continuous variables in the study

Item	NoCI	MildCI	ModerateCI	SevereCI	Total sample
Quality of life (QOL-AD NH)	38.96 ± 6.25	35.68 ± 6.83	36.24 ± 6.26	38.19 ± 6.66	37.20 ± 6.50
Depression (GDS15)	3.86 ± 3.26	5.32 ± 3.73	5.21 ± 3.76	2.74 ± 2.88	4.28 ± 3.40
Quality of life (DQOL)					
A sense of aesthetics	3.15 ± .77	3.01 ± .76	2.70 ± .62	3.22 ± .82	2.96 ± .73
Positive affect and humor	3.15 ± .77	3.01 ± .76	2.70 ± .62	3.22 ± .82	2.96 ± .73
Negative emotions	2.92 ± .82	3.19 ± .96	3.07 ± .47	2.82 ± .94	3.02 ± .75
Sense of belonging	3.00 ± .64	2.95 ± 1.12	2.85 ± .55	3.07 ± .75	2.94 ± .73
Self-esteem	3.45 ± .64	3.13 ± 1.07	2.82 ± .71	3.33 ± .77	3.12 ± .81

Table S2

Descriptive statistics for scores on QOL-AD NH items

Items	NoCI	MildCI	ModerateCI	SevereCI	Total sample
1 Physical health	2.33 ± .73	2.39 ± .67	2.30 ± .68	2.69 ± .74	2.40 ± .71
2 Vitality	2.45 ± .71	2.32 ± .70	2.26 ± .63	2.62 ± .57	2.39 ± .67
3 Moral, Mood	2.43 ± .75	2.29 ± .94	2.26 ± .69	2.50 ± .86	2.36 ± .79
4 Living environment	2.75 ± .63	2.61 ± .76	2.62 ± .67	2.65 ± .63	2.66 ± .67
5 Memory	2.57 ± .68	2.19 ± .79	2.32 ± .59	2.35 ± .75	2.36 ± .69
6 Relationships with family	3.07 ± .69	2.84 ± .99	2.72 ± .76	2.92 ± .89	2.88 ± .83
7 Relationships with staff	3.18 ± .64	2.97 ± .66	2.90 ± .58	2.81 ± .75	2.97 ± .65
8 Relationships with friends	2.72 ± .75	2.42 ± .85	2.48 ± .79	2.50 ± .65	2.54 ± .77
9 Self-image	2.30 ± .65	2.16 ± .69	2.30 ± .68	2.31 ± .74	2.27 ± .68
10 Keep busy	2.55 ± .75	2.03 ± .71	2.44 ± .61	2.46 ± .71	2.39 ± .71
11 Do things for pleasure	2.58 ± .71	2.29 ± .64	2.28 ± .73	2.50 ± .76	2.41 ± .72
12 Current life in general	2.40 ± .78	2.23 ± .76	2.34 ± .77	2.31 ± .74	2.32 ± .76
13 Ability to self-care	2.73 ± .64	2.42 ± .62	2.46 ± .58	2.54 ± .76	2.53 ± .64
14 Ability to live with others	2.50 ± .96	2.42 ± .81	2.28 ± .64	2.58 ± .64	2.42 ± .77
15 Ability to make choices	2.38 ± .84	2.10 ± .70	2.28 ± .73	2.46 ± .76	2.30 ± .76

Table S3

Pearson correlations between study variables (N = 148)

Variables	1	2	3	4	5	6	7	8	9	10	11	12
1 Age												
2 Gender	.09											
3 Marital status	**.30*****	**.30*****										
4 Level of education	.07	-.08	.06									
5 Quality of life (QoL-AD NH)	-.13	-.01	-.02	-.04								
6 Depression (GDS15)	.08	.04	-.06	-.06	**-.62*****							
7 Cognitive impairment (MMSE)	**-.19***	-.14	-.04	**.34*****	.04	.07						
8 Level of independence (GIR)	-.13	-.08	-.14	**.17***	**.17***	-.07	**.45*****					
9 DQOL sense of aesthetics	**-.26***	-.14	-.21	-.05	**.46*****	**-.36****	.09	.17				
10 DQOL positive affect	**-.26***	-.14	-.21	-.05	**.46*****	**-.36****	.09	.17	**.99*****			
11 DQOL negative affect	.01	-.13	-.15	-.09	-.06	.15	-.06	.02	-.14	-.14		
12 DQOL sense of belonging	-.11	**-.26***	-.15	.04	**.50*****	**-.30***	.08	**.32***	**.48*****	**.48*****	-.07	
13 DQOL Self-esteem	-.15	-.12	-.11	-.10	**.52*****	-.22	.22	.14	**.44*****	**.44*****	-.23	**.49*****

Note. N_{GDS} = 144. N_{GIR} = 145. N_{DQOL} = 64.

* $p < .05$. ** $p < .01$. *** $p < .001$.

Table S4

Cronbach's alpha coefficients for study variables

Variable	α
1 QOL-AD NH	.87
2 GDS-15	.70
3 DQoL Aesthetic sense	.72
4 DQoL Self-esteem	.76
5 DQoL Positive affect	.76
6 DQOL Negative affect	.85
7 DQOL sense of belonging	.52
